# Use of human PBMC to analyse the impact of obesity on lipid metabolism and metabolic status: a proof-of-concept pilot study

**DOI:** 10.1038/s41598-021-96981-6

**Published:** 2021-09-15

**Authors:** Andrea Costa, Bàrbara Reynés, Jadwiga Konieczna, Marian Martín, Miquel Fiol, Andreu Palou, Dora Romaguera, Paula Oliver

**Affiliations:** 1 Nutrigenomics, Biomarkers and Risk Evaluation (NuBE) Group, University of the Balearic Islands (UIB), Palma, Spain; 2 Research Group on Nutritional Epidemiology and Cardiovascular Physiopathology (NUTRECOR), University Hospital Son Espases (HUSE), Palma, Spain; 3 Health Research Institute of the Balearic Islands (IdISBa), Palma, Spain; 4 CIBER of Physiopathology of Obesity and Nutrition (CIBEROBN), Madrid, Spain

**Keywords:** Predictive markers, Obesity

## Abstract

Peripheral blood mononuclear cells (PBMC) are widely used as a biomarker source in nutrition/obesity studies because they reflect gene expression profiles of internal tissues. In this pilot proof-of-concept study we analysed in humans if, as we previously suggested in rodents, PBMC could be a surrogate tissue to study overweight/obesity impact on lipid metabolism. Pre-selected key lipid metabolism genes based in our previous preclinical studies were analysed in PBMC of normoglycemic normal-weight (NW), and overweight-obese (OW-OB) subjects before and after a 6-month weight-loss plan. PBMC mRNA levels of *CPT1A*, *FASN* and *SREBP-1c* increased in the OW-OB group, according with what described in liver and adipose tissue of humans with obesity. This altered expression pattern was related to increased adiposity and early signs of metabolic impairment. Greater weight loss and/or metabolic improvement as result of the intervention was related to lower *CPT1A*, *FASN* and *SREBP-1c* gene expression in an adjusted linear mixed-effects regression analysis, although no gene expression recovery was observed when considering mean comparisons. Thus, human PBMC reflect lipid metabolism expression profile of energy homeostatic tissues, and early obesity-related alterations in metabolic at-risk subjects. Further studies are needed to understand PBMC usefulness for analysis of metabolic recovery in weigh management programs.

## Introduction

Peripheral blood mononuclear cells (PBMC) are an easily obtainable blood cell fraction composed mainly by lymphocytes and monocytes^1^. Interestingly, these cells express most of the human genome, and they are able to reflect gene expression profile of key energy homeostatic tissues in response to changes in feeding conditions or to changes in body weight^1,2^. Therefore, they are increasingly being used as a source of transcriptomic biomarkers in nutrition and obesity studies^1,3^. PBMC circulate permanently through the body and are exposed to tissues with which they crosstalk to. Moreover, they respond either to internal signals (such as hormones) or external ones (such as nutrients), which can modify their gene expression profile^1,2^. Thus, PBMC act as “sentinel” cells capable of reflecting gene expression profiles of internal tissues that are more difficult to obtain, e.g. liver or adipose tissue, in response, for example, to nutritional interventions, as well as gene expression patterns characteristic of certain pathologies, e.g. obesity^2^.

Lipid metabolism has a key role in maintain energy homeostasis^4^. Excessive energy intake is properly transformed into fat in adipose tissue, and this energy store is crucial to allow survival in situations of decreased energy supply^5^. Lipid metabolism in key tissues, such as adipose tissue and liver, is altered in a situation of obesity to try to combat the excessive adiposity^6^. Our group has widely contributed to demonstrate the utility of PBMC as a surrogate tissue and as a source of biomarkers to study lipid metabolism alteration, which occurs in obesity, as well its recovery in a situation of weight loss (reviewed in^1^). Part of our expertise was developed within the FP7-European project BIOCLAIMS, coordinated by our group, dealing with the identification of new omics-derived biomarkers of health. Our previous studies have been mainly developed in rodents, which offer experimental possibilities that would be impossible to perform directly in humans. In those previous preclinical studies, using whole genome microarray analysis, we reported that lipid metabolism was the most affected pathway related to energy metabolism in PBMC of diet-induced obese rats^7,8^. Thus, we identified the interest of key lipid metabolism genes, e.g. *CPT1A*, *FASN*, *SREBP-1c*, and *PPARG*, in PBMC, as markers of adiposity-related alterations. We also confirmed the interest of these genes, in particular of *CPT1A*, in subsequent studies. We showed that their expression was altered since the first stages of obesity development or dietary unbalances conducting to increased adiposity, so they can be used as early biomarkers of adiposity-related alterations^9–12^. Moreover, impaired PBMC expression of these genes can be recovered as results of dietary interventions, so we proposed them as biomarkers of weight loss and metabolic improvement^13,14^.

With this background, in this study, we aimed to check the utility of PBMC in humans as a tool to analyse the impact of overweight and obesity on the expression of key genes of lipid metabolism. This would allow using these cells as an easily obtainable source of transcriptomic biomarkers to deepen into the knowledge of adaptations in response to increased adiposity that occur in internal homeostatic tissues, without the need of using invasive biopsies. Moreover, we aimed to analyse if PBMC are able to reflect lipid metabolism gene expression pattern recovery as result of weight loss. This could be useful to determine the effectiveness of weight-loss interventions, as the recording of the decrease in body weight and determination of the classical circulating clinical parameters, could not be reflecting metabolic adaptations occurring in relevant tissues. To fulfil our objectives, we analysed the expression of key lipid metabolism genes pre-selected based on our previous rodent studies (*CPT1A*, *FASN*, *SREBP-1c* and *PPARG*), in PBMC from normal-weight and overweight-obese subjects and in overweight-obese subjects who followed a 6-month weight-loss plan. PBMC expression patterns were correlated with relevant anthropometric and clinical parameters to establish the potential usefulness of the analysed genes as biomarkers of metabolic impairment/recovery.

Due to the increased incidence of obesity and its related morbidities, the possibility to have a non-invasive biological material, i.e. PBMC, is of great interest, given that its gene expression pattern could be easily used as an indicator of lipid metabolism alterations and as indicator of the metabolic state. This would be helpful for obesity research and could help health professionals to have an overview of the patient status in order to address more specific strategies for them, improving their overall health and preventing future diseases.

## Methods

### Experimental design

To fulfil our objectives, we conducted a pilot controlled, non-randomized, proof-of-concept trial. Young and apparently healthy subjects, including men and women were recruited and classified into two groups depending on their body mass index (BMI). A normal-weight (NW) group (BMI < 25 kg/m^2^) was composed of 20 volunteers (10 women and 10 men) and an overweight-obese (OW-OB) group (BMI ≥ 25 kg/m^2^) of 27 volunteers (13 women and 14 men). Volunteer’s flow chart is shown in Supplementary Figure S1. The inclusion criteria were: subjects aged 18–45 years old, with no chronic disease who did not take regular medication or drugs. To avoid potential bias, both groups included approx. 50% men/women and there was no difference in their average age. The number of individuals has been calculated to provide an 80% power and to detect a difference of 10% in BMI, with a p < 0.05. All the participants presented circulating glucose levels between 70 and 120 mg/dL (normoglycemic population).

Subjects from the OW-OB group followed a 6-month weight-loss program that included low-calorie food plan (30% reduction in the individual energy requirements) with dietary advice and exercise counselling. Dietary sessions were offered by a nutritionist every 15 days who provided face-to-face, individually adjusted counselling to each subject with the aim of reducing 5–10% of initial body weight. Neither dietary supplements nor vitamins were provided, and all participants consumed self-selected foods. In total, 20 out of the 27 OW-OB subjects who started the study completed the 6-month weight-loss program. Thus, these 20 subjects (9 women and 11 men) were included into an additional OW-OB subgroup, and their data were analysed after 3 and 6 months of weight loss: OW-OB-3M and OW-OB-6M subgroups.

The study protocol was approved by the Ethics Committee of Research of the Balearic Islands (CEI‐IB) (METAHEALTH-TEST; nºIB 3216/16 PI) and was carried out in accordance with The Code of Ethics of the World Medical Association (Declaration of Helsinki). Informed, written consent was obtained from all participants. Recruitment of the study subjects and data/sample collection was performed in Mallorca between 2018 and 2019. ClinicalTrials.gov Identifier: NCT04402697. Date of registration: 27/05/2020.

The primary outcome was to determine the impact of overweight/obesity on mRNA expression of selected genes on PBMC samples. Additionally, anthropometric and clinical circulating parameters were analyzed as secondary outcome indicators to understand potential correlations of PBMC gene expression with metabolic risk.

### Anthropometric, clinical and biochemical measurements

All measurements were taken at the beginning of the study in both groups (NW and OW-OB) and after 3 (OW-OB-3M) and 6 (OW-OB-6 M) months in the OW-OB group. Basic anthropometric parameters were collected: height, body weight, waist circumference and waist–hip ratio. Blood pressure was measured with a sphygmomanometer (OMRON HEM705CP, Hoofddorp, The Netherlands) after the volunteer remained seated for fifteen minutes. Additionally, hours of sleep and exercise, as well as food habits (24-h dietary recall) were recorded. Blood extraction was performed after 6 h of fast. The following circulating parameters were measured in the analytical service of the Son Espases University Hospital (HUSE) using standard procedures: plasma glucose (mg/dL), triglycerides (TG, mg/dL), total cholesterol (mg/dL), high-density lipoprotein cholesterol (HDL-C, mg/dL), gamma-glutamyl transpeptidase (GGT, U/L), and C-reactive protein (CRP, mg/dL). Low-density lipoprotein cholesterol (LDL-C, mg/dL) was calculated using the Friedewald formula whenever TG levels were inferior to 400 mg/dL^15^. Insulin levels (µU/mL) were measured with a commercial ELISA kit of Mercodia (AD bioinstruments, S.L., Terrassa, Spain).

### Indexes of clinical relevance

Subjects’ insulin resistance was assessed by the homeostatic model assessment for insulin resistance (HOMA-IR). The HOMA‐IR score was calculated from fasting insulin and glucose concentrations using the formula of Matthews et al.^16^. Additionally, the triglyceride-glucose (TyG) index was calculated as the product of fasting triglycerides and fasting glucose as it has been described as a marker to identify insulin resistance^17^. Subjects’ insulin sensitivity was assessed by the quantitative insulin sensitivity check index (QUICKI). It was calculated from fasting glucose and insulin using the formula of Katz et al.^18^. Finally, the fatty liver index (FLI) was calculated as a non-invasive method for assessing hepatic steatosis in our cohort according to Bedogni et al.^19^. It consists in an algorithm that uses waist circumference, BMI, triglycerides, and gamma-glutamyl-transferase measures. The FLI scores from 1 to 100; a score of 60 or above indicates the presence of excessive fat in the liver, although a liver biopsy is necessary to confirm the diagnose^19^.

### Body composition analysis by dual-energy X-ray absorptiometry (DXA)

Body fat was measured using a DXA scanner (GE Healthcare/DEXA Lunar Prodigy Primo; Madison, WI, USA) connected with an enCore™ software v14.10 using automatic total body scan mode. DXA systems accurately quantify the fat mass, lean mass, and bone of each segment of the body (arm, trunk, leg, android and gynoid area), and are considered a “gold standard” for body composition measurements and health assessment^20^. For visceral fat measures, scans were reanalysed using validated CoreScan software application^21^, whose algorithms work through detection of the width of the subcutaneous adipose tissue layer on the lateral part of the abdomen and the anterior–posterior thickness of the abdomen, by X-ray attenuation of the abdominal cavity in the android region. This is automated procedure developed by GE Healthcare^21^. Android-to-gynoid ratio was calculated as DXA-derived ratios of central-to-peripheral adiposity, which has been associated with risk for insulin resistance and dyslipidemia^22^.Additionally, body fat percentage was estimated using the CUN-BAE (*Clínica Universidad de Navarra*-Body Adiposity Estimator) formula^23^.

### Peripheral blood mononuclear cells isolation

Blood from 4 h fasted volunteers was collected using Vacutainer EDTA tubes. After blood collection, PBMC were isolated using Ficoll-Paque Plus density gradient media (GE Healthcare Bio Science, Madrid, Spain). In brief, the anticoagulant treated blood was diluted with an equal volume of a phosphate-buffered saline (PBS) solution. Next, the blood was layered carefully over Ficoll without intermixing (10 mL of Ficoll for 20 mL of blood mixed with PBS) in a centrifuge tub and centrifuged at 400*g* for 30 min at 20 °C in a swinging bucket rotor with acceleration and deceleration adjusted at zero. PBMC, together with platelets, were harvested from the interface between Ficoll and plasma layers. This layer was then centrifuged in PBS at 300*g* for 10 min at 20 °C to wash PBMC and to remove the platelets. PBMC pellet was finally suspended in Tripure Reagent (Roche Diagnostics, Barcelona, Spain) for its storage at – 80 °C. According to our previous evidences, approximately 84% of the cells obtained with this isolation method are lymphocytes and 16% monocytes, with no differences between NW or OW-OB subjects^24^.

### Total RNA isolation

Total RNA from PBMC samples was extracted using Tripure Reagent (Roche Diagnostics, Barcelona, Spain) and then purified with E.Z.N.A. Total RNA Kit I (Omega Biotek, Vermont, USA), following the manufacturer’s instructions. Isolated RNA was quantified using a NanoDrop ND 1000 spectrophotometer (NanoDrop Technologies, Wilmington, DE, USA). RNA integrity was confirmed using agarose gel electrophoresis.

### Real-time reverse transcriptase polymerase chain reaction (RT-qPCR)

The expression of 4 key genes involved in lipid metabolism, *CPT1A* (GenBank accession no. NM_001876.4), *FASN* (GenBank accession no. NM_004104.5), *SREBP-1c* (GenBank accession no. NM_001321096) and *PPARG* (GenBank accession no. NM_138712.5) was analysed by RT-qPCR in PBMC from all volunteers. These genes were selected based on our previous microarray analysis performed in rodents, which show that lipid metabolism genes are highly affected diet-induced obese rats^7^. Moreover, to strengthen the results, we analysed other lipid metabolism-related genes in a subset of subjects of our cohort (n = 5–9). Specifically, we analyzed *ACSL1* (GenBank accession no. NM_001286708.2), *DGAT1* (GenBank accession no. NM_012079.6), *SCD1* (GenBank accession no. NM_005063.5), *ACDVL* (GenBank accession no. NM_000018.4) and *SLC27A1* (GenBank accession no. NM_198580.3). We also analysed the proinflammatory cytokines *IL6* (GenBank accession no. NM_000600.5) and *TNF-alpha* (GenBank accession no. NM_000594.4). Equal amounts of total RNA (100 ng) were transcribed into cDNA using an iScript cDNA synthesis kit (Bio-Rad Laboratories, Madrid, Spain) in an Applied Biosystems 2720 Thermal Cycler. For each PBMC sample, three replicates were reverse-transcribed. After cDNA synthesis, real-time qPCR was performed for each RT product (three PCRs per sample) to determine mRNA expression of the selected genes. Each PCR was performed from 1/5 diluted PBMC cDNA, forward and reverse primers (5 µM), and Power SYBER Green PCR Master Mix (Applied Biosystems) in a total volume of 11 µL, with the following profile: 10 min at 95 °C, followed by a total of 40 temperature cycles (15 s at 95 °C and 1 min at 62 °C) with a final cycle of 15 s at 95 °C, 1 min at 60 °C and 15 s at 95 °C. In order to verify the purity of the products, a melting curve was produced after each run according to the manufacturer’s instructions. The threshold cycle (Ct) was calculated by the instrument’s software (StepOne Software v2.0, from Applied Biosystems), and the relative expression of each mRNA was calculated as a percentage of NW volunteers, using the 2^−ΔΔCt^ method^25^. Gene expression data were normalized against the housekeeping gene ribosomal protein, large, P0 (*RPLP0;* GenBank accession no. NM_001002.4). This gene has been previously described as a stable reference gene for human PBMC^26^. Primers sequence for the different genes are shown in Supplementary Table S1. All primers were purchased from Sigma Genosys (Sigma Aldrich Química SA, Madrid, Spain).

### Statistical analysis

Analysis was performed with SPSS for Windows v 21 (SPSS, Chicago, IL, USA). Data are expressed as mean ± SEM. Normal distribution of the data and homogeneity of variances were tested using the Shapiro–Wilk test and the Levene test, respectively. Statistical gene expression significance at the baseline point between OW-OB (with all participants; n = 27) vs NW (n = 20) was analysed using an unpaired *t* test; two-tailed. Sex differences in each of these groups was also analysed using an unpaired *t* test; two-tailed. Additionally, a repeated measures ANOVA followed by a Bonferroni post hoc test was performed within correlated samples from the OW-OB group (n = 20) that completed the 6-month weight-loss program. The differences between OW-OB, OW-OB-3M or OW-OB-6M vs NW was analysed using an unpaired *t* test; two-tailed. The same tests aforementioned above were applied to statistically compare anthropometric and serum parameters of OW-OB vs NW between both sexes and of correlated samples from the OW-OB group. A correlation between anthropometric and circulating parameters and gene expression was conducted at baseline point with OW-OB (n = 27) and NW (n = 20) participants. Pearson partial correlation coefficient was used to evaluate the relationship between gene expression analysis and the anthropometric and biochemical parameters collected at the baseline point. Partial correlation analysis was performed considering sex, and age as confounding factors; an additional partial correlation analysis including also BMI as a confounding factor was also performed. Moreover, we used an adjusted linear mixed-effects regression model with random intercepts (visit and volunteer) to assess associations between anthropometric, clinical, and biochemical parameters (exposure variables) and gene expression levels (outcome variable) during the weight loss intervention, using repeated measurements at three time points (3 visits: basal, 3M and 6M); the model was adjusted for sex and age. Threshold of significance was defined at p < 0.05 for all analysis. Missing data (serum parameters of just one NW subject) were not included in the analysis.

A principal component analysis (PCA) was performed after data normalization using MetaboAnalyst 3.0 software^27^. All the anthropometric and biochemical parameters, as well as mRNA expression data of the key genes analyzed, *CPT1A*, *FASN*, *SREBP-1c* and *PPARG* (29 variables in total) were included in the analysis. The score plot of PC1 versus PC2 was used to examine the potential contribution of these components to discriminate the groups of our cohort. Gene expression data of the following genes were not included as they were analyzed only in a subset of subjects of our cohort: *ACSL1, DGAT1, SCD1, ACADVL, SLC27A1, IL-6* and *TNF-alpha.*

## Results

### Normal-weight and overweight-obese subject’s characteristics at baseline point

Volunteers were extensively characterised to be able to compare if gene expression changes analysed in this study correlated with the most classical biomarkers of metabolic risk. The anthropometric measurements, body fat content, relevant circulating parameters and clinical indexes of the participants at the beginning of the study are presented in Tables 1 and 2. Data are presented including both sexes, as well as stratified by sex.

**Table 1:** Anthropometric measurements, body composition, and blood pressure in the NW and OW-OB groups.

	Normal-weight (NW)			Overweight-obese (OW-OB)		
	Women and men	Women	Men	Women and men	Women	Men
**Anthropometric measurements**						
Number of volunteers	20	10	10	27	13	14
Age (years)	29.0 ± 1.7	29.0 ± 1.6	29.0 ± 2.4	32.0 ± 1.4	31.0 ± 2.0	33.0 ± 1.5
Weight (kg)	66.7 ± 2.3	59.8 ± 1.8	73.7 ± 2.9^#^	98.2 ± 3.4*	90.7 ± 4.6	105 ± 4^#^
Height (cm)	173 ± 0	165 ± 0	180 ± 0^#^	170 ± 0	164 ± 0	175 ± 0^#^
BMI (kg/m^2^)	22.3 ± 0.5	21.9 ± 0.6	22.8 ± 0.7	34.0 ± 1.0*	33.7 ± 1.8	34.2 ± 1.1
Waist-hip ratio	0.79 ± 0.01	0.74 ± 0.02	0.84 ± 0.01^#^	0.88 ± 0.02*	0.80 ± 0.02	0.96 ± 0.02^#^
Waist circumference (cm)	76.5 ± 1.6	71.4 ± 1.4	81.6 ± 1.7^#^	100 ± 3*	93.7 ± 3.5	107 ± 3^#^
**Body composition**						
DXA (% body fat)	27.2 ± 2.0	34.2 ± 1.6	20.1 ± 2.0^#^	40.2 ± 1.5*	46.0 ± 1.9	35.1 ± 1.1^#^
DXA (% visceral fat)	0.35 ± 0.07	0.27 ± 0.06	0.45 ± 0.11	1.27 ± 0.13*	0.97 ± 0.17	1.54 ± 0.18^#^
DXA (kg android fat)	1.13 ± 0.12	1.30 ± 0.14	0.96 ± 0.20	3.81 ± 0.29*	3.51 ± 0.46	4.09 ± 0.37
DXA (kg gynoid fat)	3.29 ± 0.29	4.11 ± 0.26	2.47 ± 0.39^#^	6.41 ± 0.41*	7.48 ± 0.67	5.42 ± 0.30^#^
Android-to-gynoid ratio	0.34 ± 0.02	0.31 ± 0.02	0.37 ± 0.04	0.61 ± 0.04*	0.46 ± 0.04	0.75 ± 0.04^#^
CUN-BAE (% body fat)	23.6 ± 1.5	28.9 ± 1.2	18.2 ± 1.1	39.6 ± 1.5*	45.0 ± 1.9	35.1 ± 1.3^#^
Lean mass (%)	68.8 ± 2.0	61.9 ± 1.5	75.6 ± 1.9^#^	56.8 ± 1.4*	51.6 ± 1.8	61.6 ± 1.1^#^
**Blood pressure**						
SBP (mmHg)	123 ± 3	117 ± 3	129 ± 4^#^	128 ± 2	121 ± 3	136 ± 3^#^
DBP (mmHg)	73.0 ± 1.3	73.0 ± 1.7	73.0 ± 1.9	77.5 ± 3.2	80.0 ± 2.3	74.5 ± 6.0

Data in each group (NW and OW-OB) is analysed considering all the individuals (women and men together), and stratified by sex. Data are mean ± SEM (n = 20 in the NW and n = 27 in the OW-OB group). Statistics: * OW-OB vs NW, and^#^ NW men vs NW woman and OW-OB men vs OW-OB woman, p < 0.05, Student’s *t* test.

**Table 2:** Circulating parameters and indexes of clinical relevance in NW and OW-OB groups.

	Normal-weight (NW)			Overweight-obese (OW-OB)		
	Women and men	Women	Men	Women and men	Women	Men
**Circulating parameters**						
Number of volunteers	19	10	9	27	13	14
Glucose (mg/dL)	84.2 ± 1.3	83.0 ± 1.8	85.6 ± 2.02	81.0 ± 1.2	79.3 ± 1.7	82.7 ± 1.6
Insulin (µU/mL)	3.91 ± 0.32	4.33 ± 0.40	3.50 ± 0.50	7.47 ± 0.70*	7.29 ± 1.05	7.64 ± 0.98
Triglycerides (mg/dL)	65.9 ± 6.5	70.1 ± 9.5	61.2 ± 9.1	102 ± 10*	90.4 ± 7.7	113 ± 16
Total cholesterol (mg/dL)	164 ± 10	171.0 ± 12.8	156.4 ± 16.6	193 ± 6*	192.7 ± 5.8	193.3 ± 11.0
LDL-C (mg/dL)	91.7 ± 6.8	92.4 ± 8.0	90.9 ± 11.8	117 ± 5*	112 ± 6	121 ± 8
HDL-C (mg/dL)	59.2 ± 3.8	64.6 ± 5.3	53.3 ± 5.1	56.0 ± 2.0	62.7 ± 3.1	50.2 ± 1.5^#^
Gamma-glutamyl transferase (U/L)	25.6 ± 1.8	23.1 ± 1.5	28.3 ± 3.2	37.1 ± 2.7*	29.0 ± 2.2	45.9 ± 3.6^#^
C-reactive protein (mg/dL)	0.09 ± 0.03	0.13 ± 0.05	0.04 ± 0.01	0.43 ± 0.11*	0.51 ± 0.21	0.34 ± 0.09
**Indexes of clinical relevance**						
HOMA-IR	0.83 ± 0.08	0.89 ± 0.09	0.77 ± 0.13	1.52 ± 0.16*	1.46 ± 0.21	1.58 ± 0.25
Triglyceride-glucose index	7.85 ± 0.09	7.90 ± 0.11	7.79 ± 0.14	8.24 ± 0.09*	8.14 ± 0.09	8.33 ± 0.15
QUICKI	0.40 ± 0.01	0.40 ± 0.01	0.41 ± 0.01	0.37 ± 0.01*	0.37 ± 0.01	0.37 ± 0.01
Fatty liver index	10.2 ± 1.9	6.91 ± 0.97	13.8 ± 3.6	68.0 ± 6.2*	55.1 ± 9.1	82.0 ± 6.2^#^

Data in each group (NW and OW-OB) is analysed considering all the individuals (women and men together), and stratified by sex. Data are mean ± SEM (n = 20 in the NW and n = 27 in the OW-OB group). Statistics: *OW-OB vs NW, and ^#^NW men vs NW woman and OW-OB men vs OW-OB woman, p < 0.05, Student’s *t* test. Data of circulating parameters in the NW group correspond to 19 subjects because of missing data of one volunteer.

As shown in Table 1, subjects included in the two studied groups (NW and OW-OB), with n = 20 and 27, respectively, did not differ in age or in blood pressure, but body weight, BMI, waist-hip ratio, waist circumference and body fat content estimated using the CUN-BAE formula were higher in the OW-OB group. OW-OB subjects also presented greater percentage of body fat obtained from DXA values, including greater amount of visceral, android and gynoid fat, as well as greater android-to-gynoid ratio, but lower lean mass than NW. When discriminating by sex within the respective body mass groups (NW and OW-OB), we found that men presented higher values than women for the following anthropometric measurements: body weight, height, waist-hip ratio, waist circumference, lean mass and systolic blood pressure. However, both NW and OW-OB men presented lower DXA-derived body fat percentage and lower gynoid fat than NW and OW-OB women. Nevertheless, visceral fat and android-to-gynoid ratio was greater in OW-OB men than in women.

Regarding circulating parameters (Table 2), although OW-OB subjects did not present altered glucose levels, they presented signs of insulin resistance, such as increased insulin levels and alterations in insulin resistance parameters: HOMA‐IR, triglyceride-glucose index and QUICKI. Moreover, the OW-OB group presented higher levels of circulating triglycerides, total cholesterol, LDL-cholesterol, gamma-glutamyl transferase and C-reactive protein; although they were all within accepted clinical reference intervals. No difference was observed for HDL-cholesterol levels. Regarding the fatty liver index, the OW-OB group exhibited a score of 68.0 vs 10.2 in the NW group, which is considered as suggestive of fatty liver. When discriminating by sex, OW-OB men presented greater gamma-glutamyl transferase levels than OW-OB women, while HDL-cholesterol values were higher in OW-OB women. Additionally, OW-OB men presented a higher fatty liver index than OW-OB women (82.0 vs 55.1), which suggests, in this cohort, that men are more susceptible to develop fatty liver.

### Overweight-obese subject’s characteristics during the 6-month weight-loss plan

Anthropometric measurements, body fat content, relevant circulating parameters and clinical indexes of the OW-OB participants who finished the 6-month weight-loss plan are presented in Tables 3 and 4. To check the effects of the weight-loss program only those individuals who completed the 6-month intervention were selected, i.e. 20 out of 27 initial participants. Volunteers who quit the trial before completing the 6-month intervention was because their incompatibility to attend medical visits of the study. Data were collected at baseline point and after 3 and 6 months of intervention. The intervention produced a moderate but considerable weight loss, 3.34% after 3 months and 4.28% after 6 months (Table 3). This lower body weight was accompanied by a lower waist circumference, lower amount of android and gynoid fat as well as lower DXA-fat percentage (2.81% and 5.12% of decrease, after 3 and 6 months respectively), while a substantial increase in lean mass was observed (1,73% and 3.11%, after 3 and 6 months). However, visceral fat loss at the end of the intervention (8.62%) did not reach statistical significance. In spite of the modest body weight and body fat loss, metabolic recovery was observed. Circulating levels of triglycerides, total cholesterol, LDL-cholesterol and gamma-glutamyl transferase decreased already after 3 months of intervention, reaching the same levels as those in the NW group (Table 4). These parameters increased after 3 additional months of intervention (OW-OB-6M group), although they continued to be lower than in the basal OW-OB point, and not different from values of the NW volunteers. A recovery to levels of the NW group was observed also for the triglyceride-glucose index. Moreover, circulating glucose levels were lower after 3 and 6 months of the intervention in comparison to levels in the NW group. An improvement during the intervention was also observed for HOMA-IR, and fatty liver indexes, although in this case, values remained higher than in NW volunteers at the end of the intervention period.

**Table 3:** Anthropometric data, body composition and blood pressure in overweight-obese participants that completed the 6-month intervention.

	Normal-weight (NW)	Overweight-obese (OW-OB)	OW-OB-3M	OW-OB-6M
**Anthropometric measurements**				
Number of volunteers	20	20	20	20
Age (years)	29.0 ± 1.7	31.0 ± 1.4	31.0 ± 1.4	31.0 ± 1.4
Weight (kg)	66.7 ± 2.3	95.9 ± 3.9*a	92.7 ± 3.6*b	91.8 ± 3.7*b
BMI (kg/m^2^)	22.3 ± 0.4	34.3 ± 1.30*a	32.1 ± 1.3*b	31.8 ± 1.0*b
Waist-hip ratio	0.79 ± 0.01	0.88 ± 0.02*	0.88 ± 0.02*	0.88 ± 0.01*
Waist circumference (cm)	76.5 ± 1.6	98.9 ± 2.7*a	95.7 ± 2.4*b	95.2 ± 2.5*b
**Body composition**				
DXA (% body fat)	27.1 ± 2.0	39.1 ± 1.8*a	38.0 ± 1.9*b	37.1 ± 1.9*c
DXA (% visceral fat)	0.35 ± 0.07	1.16 ± 0.12*	1.04 ± 0.11*	1.06 ± 0.13*
DXA (kg android fat)	1.13 ± 0.12	3.53 ± 0.30*a	3.24 ± 0.29*b	3.13 ± 0.30*b
DXA (kg gynoid fat)	3.29 ± 0.29	6.14 ± 0.49*a	5.70 ± 0.41*b	5.46 ± 0.44*b
Android-to-gynoid ratio	0.34 ± 0.02	0.60 ± 0.04*	0.60 ± 0.04*	0.59 ± 0.04*
CUN-BAE (% body fat)	23.6 ± 1.5	38.3 ± 1.7*a	37.2 ± 1.8*b	37.0 ± 1.8*b
Lean mass (%)	68.8 ± 2.0	57.8 ± 1.7*a	58.8 ± 1.7*b	59.6 ± 1.8*c
**Blood pressure**				
SBP (mmHg)	123 ± 3	126 ± 3a	119 ± 4a,b	119 ± 3b
DBP (mmHg)	73.0 ± 1.3	80.0 ± 1.5a	78.0 ± 3*a,b	73.0 ± 2.6b

Data are mean ± SEM (n = 20 in the NW and n = 20 in the different OW-OB groups). Statistics: within the OW-OB groups, values not sharing a common letter (a, b) are significantly different (repeated measures ANOVA, p < 0.05); no letter indicates no significant differences; *indicates values significantly different vs NW group (Student’s *t* test, p < 0.05).

**Table 4:** Circulating parameters and indexes of clinical relevance in overweight-obese participants that completed the 6-month intervention.

	Normal-weight (NW)	Overweight-obese (OW-OB)	OW-OB-3M	OW-OB-6M
**Circulating parameters**				
Number of volunteers	19	20	20	20
Glucose (mg/dL)	84.2 ± 1.3	80.1 ± 1.1	80.0 ± 1.4*	75.6 ± 2.4*
Insulin (µU/mL)	3.91 ± 0.32	6.80 ± 0.70*a	5.84 ± 0.58*a	5.15 ± 0.48*b
Triglycerides (mg/dL)	65.9 ± 6.5	95.2 ± 10.9*a	68.8 ± 7.9 b	80.3 ± 11.1a,b
Total cholesterol (mg/dL)	164 ± 10	190 ± 8*a	144 ± 9 b	173 ± 8c
LDL-C (mg/dL)	91.7 ± 6.8	114 ± 6*a	85.5 ± 7.0 b	102 ± 6c
HDL-C (mg/dL)	59.2 ± 3.8	57.1 ± 2.6a	44.5 ± 2.3*b	54.3 ± 2.5a
Gamma-glutamyl transferase (U/L)	25.6 ± 1.8	36.6 ± 3.0*a	22.6 ± 2.8b	28.7 ± 2.8b
C-reactive protein (mg/dL)	0.09 ± 0.03	0.46 ± 0.15*	0.45 ± 0.13*	0.33 ± 0.10*
**Indexes of clinical relevance**				
HOMA-IR	0.83 ± 0.08	1.34 ± 0.15*a	1.37 ± 0.14*a	1.12 ± 0.11*b
Triglyceride-glucose index	7.85 ± 0.09	8.15 ± 0.11*a	7.80 ± 0.11b	7.90 ± 0.11b
QUICKI	0.40 ± 0.01	0.38 ± 0.01*	0.37 ± 0.01*	0.38 ± 0.01*
Fatty liver index	10.2 ± 1.9	64.7 ± 7.0*a	47.5 ± 7.3*b	52.3 ± 6.1*b

Data are mean ± S.E.M (n = 20 in the NW and n = 20 in the different OW-OB groups). Statistics: within the OW-OB groups, values not sharing a common letter (a, b) are significantly different (repeated measures ANOVA, p < 0.05); no letter indicates no significant differences; * indicates values significantly different vs NW group (Student’s *t* test, p < 0.05). Data of circulating parameters in the NW group correspond to 19 subjects because of missing data of one volunteer.

### Expression of key lipid metabolism genes in PBMC from NW and OW-OB subjects and effect of the 6-month weight-loss plan on their expression in OW-OB subjects

Four key genes involved in lipid metabolism were selected to be analysed at mRNA expression level in PBMC from our volunteers. Genes were: *CPT1A*, which plays an important role in fatty acid beta-oxidation in the mitochondria; the lipogenic *FASN* and *SREBP-1c* genes, involved in the formation of long-chain fatty acids; and *PPARG*, coding for a key adipogenesis regulator. These genes were selected based on our previous preclinical studies using rodents, showing that these genes are expressed in detectable amounts in PBMC, and are regulated in these cells in the same way as in internal tissues with a fundamental role in energy homeostasis, such as liver and adipose tissue (reviewed in^1^).

**Figure 1:**
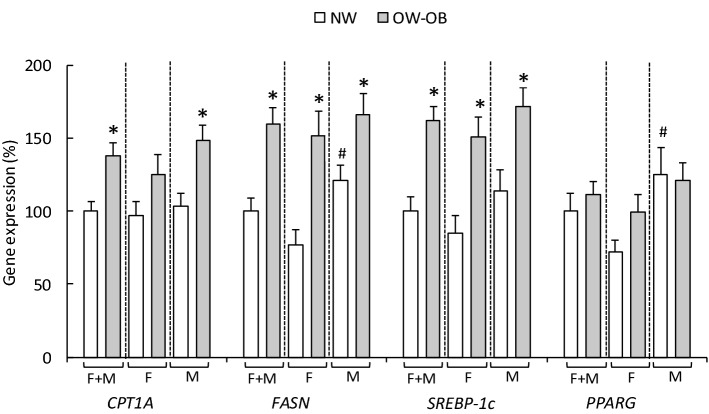
Expression of lipid metabolism genes in PBMC from NW and OW-OB subjects and stratified by sex in each of the groups. Data are mean ± SEM (n = 20 in the NW and n = 27 in the OW-OB group) and are expressed as a percentage of the mean value of the NW group. Statistics: *OW-OB vs NW;^#^NW men vs NW woman, and OW-OB men vs OW-OB woman (p < 0.05, Student’s *t* test; two tailed; unpaired samples).

As presented in Fig. 1, gene expression of *CPT1A*, *FASN* and *SREBP-1c* was increased in PBMC of the OW-OB group. This increased expression as result of overweight and obesity was observed even after splitting by sex for *FASN* and *SREBP-1c*. For *CPT1A*, mRNA expression was also greater in OW-OB than in NW men, but in women the increase was not statistically significant due to variability (p = 0.193). Additionally, results also made evident that NW men presented higher gene expression levels of *FASN* and *PPARG* than those of NW women.

**Figure 2:**
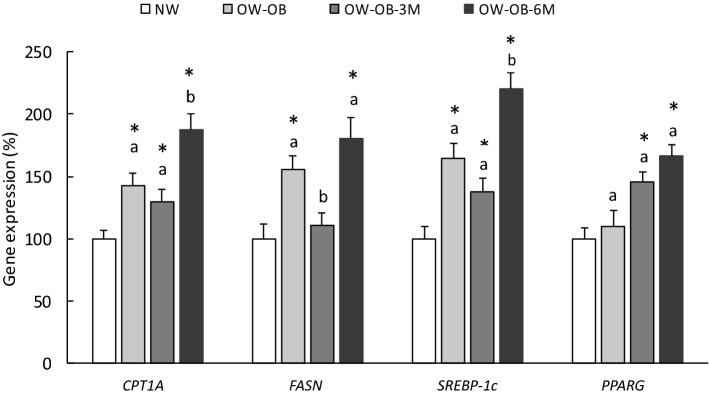
Expression of lipid metabolism genes in PBMC from NW and OW-OB subjects who completed the weight-loss intervention, at 3 and 6 months (OW-OB-3M and OW-OB-6M). Data are mean ± SEM (n = 20 in the NW and n = 20 in the different OW-OB groups) and are expressed as a percentage of the mean value of the NW group. Statistics: within the OW-OB groups, values not sharing a common letter (**a**,**b**) are significantly different (repeated measures ANOVA, p < 0.05); no letter indicates no significant differences; * indicates values significantly different vs NW group (p < 0.05, Student’s *t* test; two tailed; unpaired samples).

Subsequently, in order to analyse the impact of the weight-loss intervention on PBMC gene expression, we focused our study in those OW-OB volunteers (n = 20 out of 27) that completed the intervention (Fig. 2). In this case, analyses were performed without separating by sex because, as seen previously, no relevant variances at gene expression level were evident in the OW-OB group. At the baseline point, we found the same differences between OW-OB vs NW as when we considered all the participants of the OW-OB group, i.e. increased expression levels of *CPT1A*, *FASN* and *SREBP-1c*, confirming the robustness of this observation. After 3 months of dietary intervention, we observed a recovery that was only evident for PBMC *FASN* expression, whose levels decreased reaching the values observed in PBMC from NW subjects. However, no significant changes were observed for gene expression of *CPT1A* or *SREBP-1c* at this time-point. Surprisingly, after 6 months, gene expression of *CPT1A*, *FASN* and *SREBP-1c* was increased in comparison to the levels observed at 3 months. Regarding *PPARG*, its expression, which was not affected in the OW-OB group at basal point, was increased after 3 and 6 months of intervention.

In order to strengthen these findings, additional genes selected based on their relevance in lipid metabolism were analysed: *ACSL1* that plays a role in the activation of long-chain fatty acids to acyl-CoAs to enter both in lipid synthesis or degradation pathways; *DGAT1*, involved in the synthesis of triacylglycerols from diacylglycerols; *SCD1*, involved in the synthesis of unsaturated fatty acids; *ACADVL* that codes for one of the enzymes that catalyses the first step of mitochondrial fatty acid beta-oxidation, and *SLC27A1*, which codes for fatty acid transport protein 1. These additional genes were examined in a subset of subjects of our cohort (NW, n = 5; OW-OB, n = 9; OW-OB-3M, n = 6 and OW-OB-6M, n = 6). With these more limited number of samples, we observed a trend for a decrease in basal gene expression of *SCD1* in the OW-OB group (73.3 ± 7.3 in the OW-OB vs 100 ± 10 in the NW group) (p < 0.1, Mann–Whitney U test), which was normalized to that of NW animals at the end of the weight-loss intervention (OW-OB-6M), as well as a significant decreased expression of *SLC27A1* in the OW-OB group (63.7 ± 11.8 in the OW-OB vs 100 ± 11) (p < 0.05, Mann–Whitney U test). No significant changes were observed for the expression of *ACSL1*, *DGAT1* or *ACDVL.* Because obesity is related to chronic low grade inflammation, we also analysed PBMC mRNA expression of the proinflammatory genes *IL6* and *TNF-alpha*, which was not different between the NW and OW-OB groups. As has been previously described in humans, there is a lack of correlation between altered serum IL6 and TNF-alpha levels and altered PBMC gene expression^28^. Thus, PBMC do not appear to contribute to increased cytokine levels found in obesity, which would derive mainly from adipocytes with an excessive amount of fat.

### Principal component analysis (PCA)

In order to study which of the all variables collected most strongly describe the different groups of our cohort a PCA was performed after data normalization. When we performed the analysis with the NW and OW-OB groups, a clear clustering of the subjects was observed (Supplementary Figure S2A,B). The first and second components (PC1 and PC2) explained 53% of the total variance. PC1 (41.5% of the variance) was mainly characterized by kg of android fat (0.285), fatty liver index (0.271), BMI (0.266), body weight (0.266) and % of visceral fat (0.260). Concerning PC2 (11.5%), the main variables were waist-hip ratio (0.401), android-to-gynoid ratio (0.206), % of body fat measured by DXA (− 0.443), kg gynoid fat (− 0.415), and % body fat measured by CUN-BAE (− 0.300). Thus, the parameters that showed higher importance in the separation of the groups were anthropometric parameters. Gene expression of *CPT1A*, *FASN*, *SREBP-1c* and *PPARG* had a global weight of 3.9% (out of 53% of total variance), with *FASN* as the one with the highest contribution in the clustering.

When we performed the analysis taking into the account the four groups of the study (NW, OW-OB, OW-OB-3M and OW-OB-6M), the 3 OW-OB groups were not capable of being mathematically distinguished between them on the basis of the data collected (Supplementary Figure S2C). Although biochemical parameters were improved and even in some cases normalized to those of the NW group, weight-loss intervention produced only a moderate decrease in body weight and fat content, which are those parameters with a higher loading weight in the clustering. This could explain that no clear separation was observed for the subjects with OW-OB before, or after 3 (OW-OB-3M) or 6 (OW-OB-6M) months of intervention, as the differences between these three groups are less prominent compared to those with the NW group. In spite of this, a higher overlapping was observed within the OW-OB-3M and OW-OB-6M groups with the NW group, which is indicative of the effectiveness of the weight-loss intervention.

### Correlation of PBMC gene expression with different anthropometric, clinical and biochemical parameters

We were interested to assess if altered gene expression pattern of key genes of lipid metabolism observed in PBMC could be useful as a biomarker of metabolic impairment related to overweight and obesity, and/or its recovery after weight loss. Thus, we performed partial correlation analyses with different anthropometric, clinical and biochemical parameters (Table 5). When considering together NW (n = 20) and all OW-OB (n = 27) participants at the beginning of the study, and sex and age as confounding factors, relevant associations were observed. PBMC gene expression of *CPT1A*, *FASN* and *SREBP-1c* was directly correlated (p < 0.05) with body weight, waist-hip ratio, waist circumference, % of body fat estimated by CUN-BAE and fatty liver index. Expression of the lipogenic genes *FASN* and *SREBP-1c* also correlated directly (p < 0.05) with BMI and circulating TG, and *SREBP-1c* with gynoid and android fat mass, and with circulating levels of CRP and GGT. When a trend of significance (p < 0.1) was considered, additional correlations were observed: *CPT1A* expression correlated directly with, android fat, android-to-gynoid ratio, circulating TG and LDL-cholesterol levels and with triglyceride-glucose index; *FASN* with android and gynoid fat and triglyceride-glucose index; and *SREBP-1c* with visceral fat, android-to-gynoid ratio, circulating insulin, triglyceride-glucose index and with systolic blood pressure. Moreover, a trend for an inverse correlation (p < 0.1) was observed between the lipogenic genes *FASN* and *SREBP-1c* and lean mass. In the case of *PPARG*, its gene expression levels in PBMC did not correlate with any of the analysed parameters. We also performed partial correlation analyses, adjusted by sex, age and including BMI. After including BMI as confounding factor, some of the associations were maintained: *CPT1A* correlated directly (p < 0.05) with weight and waist-hip ratio; and *SREBP-1c* mRNA expression with waist-hip ratio and fatty liver index. However, the associations with circulating parameters disappeared.

**Table 5:** Partial correlation of lipid metabolism genes with anthropometric, clinical and biochemical parameters from all the volunteers (NW and OW-OB), adjusted by sex and age.

Anthropometric measurements	*CPT1A*		*FASN*		*SREBP-1c*		*PPARG*	
	R	p	R	p	R	p	R	p
Weight (kg)	0.362*	0.032	0.397*	0.018	0.470**	0.004	0.090	0.607
BMI (kg/m^2^)	0.264	0.126	0.347*	0.041	0.420*	0.012	0.176	0.313
Waist-hip-ratio	0.479**	0.004	0.401*	0.017	0.521**	0.001	0.091	0.605
Waist circumference (cm)	0.317*	0.043	0.389*	0.012	0.439**	0.004	0.075	0.639
**Body composition**								
DXA (% body fat)	0.222	0.200	0.203	0.229	0.266	0.123	0.102	0.560
DXA (% visceral fat)	0.238	0.168	0.281	0.101	0.333^#^	0.050	0.176	0.311
DXA (kg android fat)	0.324^#^	0.057	0.308^#^	0.072	0.343*	0.043	0.096	0.582
DXA (kg gynoid fat)	0.210	0.226	0.322^#^	0.059	0.351*	0.039	0.076	0.664
Android-to-gynoid ratio	0.309^#^	0.071	0.215	0.214	0.326^#^	0.056	0.077	0.660
CUN-BAE (% body fat)	0.322*	0.040	0.399*	0.010	0.439**	0.004	0.098	0.541
Lean mass (% body fat)	− 0.241	0.130	− 0.294^#^	0.062	− 0.307^#^	0.051	− 0.055	0.733
**Circulating parameters**								
Glucose (mg/dL)	− 0.219	0.129	− 0.017	0.922	− 0.187	0.282	0.105	0.547
Insulin (µU/mL)	0.214	0.216	0.253	0.143	0.330^#^	0.053	− 0.073	0.679
Triglycerides (mg/dL)	0.322^#^	0.059	0.361*	0.033	0.377*	0.026	0.027	0.878
Total cholesterol (mg/dL)	0.282	0.101	0.193	0.266	0.257	0.136	0.004	0.982
LDL-C (mg/dL)	0.307^#^	0.073	0.166	0.340	0.215	0.214	0.019	0.912
HDL-C (mg/dL)	− 0.044	0.803	− 0.031	0.860	0.053	0.764	− 0.054	0.758
Gamma-glutamyl transferase (U/L)	0.262	0.129	0.153	0.379	0.395*	0.019	0.066	0.704
C-reactive protein (mg/dL)	0.108	0.536	0.134	0.442	0.338*	0.047	0.079	0.653
**Indexes of clinical relevance**								
HOMA-IR	0.197	0.257	0.247	0.152	0.287	0.094	− 0.082	0.641
Triglyceride-glucose index	0.298^#^	0.082	0.331^#^	0.052	0.315^#^	0.065	0.103	0.556
QUICKI	− 0.092	0.598	− 0.196	0.260	− 0.249	0.149	− 0.043	0.805
Fatty liver index	0.336*	0.049	0.393*	0.019	0.534**	0.001	0.185	0.287
**Blood pressure**								
SBP (mmHg)	0.163	0.351	0.226	0.192	0.313^#^	0.067	0.074	0.673
DBP (mmHg)	− 0.175	0.313	− 0.001	0.997	0.171	0.327	0.091	0.601

R = Pearson correlation coefficients and p values associated to each correlation. *p < 0.05, **p < 0.01 and ^#^p < 0.1. NW (n = 20) and OW-OB (n = 27) data from the baseline point were used.

Finally, we analysed associations between gene expression and anthropometric, clinical, and biochemical parameters during the 6-month weight-loss intervention (OW-OB, OW-OB-3M and OW-OB-6M), using a linear mixed-effects regression model adjusted for sex and age (Table 6). Associations found in the intervention were in line with those obtained using data from NW and OW-OB groups at the beginning of the study. *CPT1A* gene expression levels were associated with greater % of body fat measured by DXA, diastolic blood pressure and fatty liver index values, and with lower lean mass. In the same trend, *FASN* gene expression levels were associated with greater total cholesterol and LDL-cholesterol levels, and *SREBP-1c* expression with greater circulating gamma-glutamyl transferase levels. When a trend of significance (p < 0.1) was considered, *CPT1A* expression also correlated directly with waist circumference, android fat, android-to-gynoid ratio, and % fat measured by CUN-BAE.

**Table 6:** Associations between concurrent 6-month weight loss-intervention (OW-OB, OW-OB-3M and OW-OB-6M groups) in gene expression levels (outcome variable) and anthropometric, clinical, and biochemical parameters (exposure variable), adjusted by age and sex.

	*CPT1A*		*FASN*		*SREBP-1c*		*PPARG*	
	β (95% CI)	p	β (95% CI)	p	β (95% CI)	p	β (95% CI)	p
**Anthropometric measurements**								
Weight (kg)	0.498 (− 0.392; 1.39)	0.255	− 0.784 (− 1.75; 0.184)	0.106	− 0.949 (− 2.18; 0.287)	0.124	0.451 (− 1.05; 1.95)	0.534
BMI (kg/m^2^)	2.07 (− 0.681; 4.82)	0.131	− 1.42 (− 4.71; 1.87)	0.376	− 2.40 (− 6.48; 1.67)	0.232	1.73 (− 2.89; 6.36)	0.439
Waist circumference (cm)	1.19^#^ (− 0.224; 2.60)	0.094	− 0.157 (− 1.86; 1.54)	0.848	− 0.662 (− 2.75; 1.43)	0.516	0.672 (− 1.63; 2.97)	0.548
Waist-hip-ratio	21.3 (− 258; 301)	0.879	179 (− 113; 470)	0.225	33.3 (− 300; 367)	0.842	26.6 (− 342; 392)	0.893
**Body composition**								
DXA (% body fat)	2.44* (0.002; 4.89)	0.050	− 0.641 (− 3.58; 2.29)	0.654	− 2.16 (− 5.69; 1.36)	0.215	2.16 (− 1.56; 5.88)	0.240
DXA (% visceral fat)	18.0 (− 9.15; 45.1)	0.181	10.1 (− 22.0; 42.2)	0.519	3.86 (− 36.1; 43.9)	0.843	12.0 (− 29.5; 53.5)	0.552
DXA (kg android fat)	9.60^#^ (− 0.301; 19.5)	0.057	− 2.03 (− 14.4; 10.3)	0.733	− 6.67 (− 21.8; 8.45)	0.367	8.56 (− 7.50; 24.6)	0.277
DXA (kg gynoid fat)	3.65 (− 5.17; 12.5)	0.400	− 7.33 (− 16.8; 2.17)	0.124	− 9.26 (− 21.1; 2.61)	0.120	6.67 (− 6.59; 19.2)	0.308
Android-Gynoid ratio	92.1^#^ (− 3.02; 187)	0.057	35.9 (− 79.6; 151)	0.523	33.8 (− 111; 179)	0.633	28.2 (− 120; 177)	0.698
CUN-BAE (% body fat)	2.06^#^ (− 0.245; 4.37)	0.076	− 1.21 (− 3.91; 1.49)	0.358	− 2.28 (− 5.70; 1.14)	0.177	1.86 (− 2.06; 5.79)	0.329
Lean mass (% body fat)	− 2.75* (− 5.32; − 0.184)	0.037	− 0.626 (− 2.43; 3.68)	0.672	2.60 (− 1.13; 6.33)	0.160	− 3.12 (− 6.96; 0.724)	0.105
**Blood pressure**								
SBP (mmHg)	− 0.424 (− 1.50; 0.653)	0.431	0.309 (− 0.762; 1.38)	0.563	− 1.02 (− 1.66; − 0.372)	0.922	0.350 (− 103; 1.73)	0.612
DBP (mmHg)	1.39* (0.037; 2.74)	0.044	0.530 (− 0.861; 1.92)	0.448	0.858 (− 0.769; 2.49)	0.295	1.21 (− 0.591; 3.00)	0.183
**Circulating parameters**								
Glucose (mg/dL)	1.31 (− 0.446; 3.09)	0.140	0.138 (− 1.67; 1.95)	0.879	0.528 (− 1.47; 2.53)	0.598	− 1.97 (− 4.24; 0.294)	0.087
Insulin (µU/mL)	− 0.258 (− 5.26; 4.74)	0.917	3.15 (− 2.14; 8.44)	0.234	2.67 (− 3.76; 9.10)	0.406	0.183 (− 6.91; 7.27)	0.958
Triglycerides (mg/dL)	0.134 (− 0.167; 0.436)	0.369	0.134 (− 0.178; 0.445)	0.386	0.268 (− 0.121; 0.658)	0.171	− 0.416 (− 0.841; 0.009)	0.055#
Total cholesterol (mg/dL)	0.124 (− 0.241; 0.490)	0.494	0.361* (0.010; 0.712)	0.044	0.203 (− 0.252; 0.658)	0.374	− 0.553* (− 1.04; − 0.061)	0.029
LDL-C (mg/dL)	0.175 (− 0.327; 0.677)	0.482	0.502* (0.032; 0.971)	0.037	0.341 (− 0.311; 0.994)	0.296	− 0.645^#^ (− 1.35; 0.063)	0.073
HDL-C (mg/dL)	− 0.117 (− 1.49; 1.26)	0.865	0.571 (− 0.832; 1.97)	0.418	− 0.411 (− 1.97; 1.15)	0.598	− 1.41 (− 3.21; 0.396)	0.123
C-reactive protein (mg/dL)	9.60 (− 15.7; 34.9)	0.438	− 8.39 (− 35.9; 19.1)	0.534	8.97 (− 25.8; 43.7)	0.600	8.99 (− 24.4; 42.3)	0.581
Gamma-glutamyl transferase (U/L)	− 0.978 (− 0.358; − 2.31)	0.145	0.800 (− 0.610; 2.21)	0.257	2.10* (0.473; 3.72)	0.013	− 0.630 (− 2.55; 1.29)	0.509
**Indexes of clinical relevance**								
HOMA-IR	3.49 (− 21.1; 28.0)	0.774	17.4 (− 7.57; 42.4)	0.166	12.6 (− 18.3; 43.5)	0.415	− 11.9 (− 47.0; 23.2)	0.495
QUICKI	− 212 (− 713; 288)	0.391	− 361 (− 881; 158)	0.166	− 332 (− 981; 316)	0.306	23.5 (− 678; 725)	0.946
Triglyceride-glucose index	21.0 (− 6.31; 48.4)	0.127	5.79 (− 23.5; 35.1)	0.690	26.3 (− 8.18; 60.8)	0.131	− 50.7 (− 89.1; − 12.5)	0.011
Fatty liver index	0.608* (0.139; 1.07)	0.013	− 0.106 (− 0.670; 0.458)	0.699	0.126 (− 0.639; 0.890)	0.737	− 0.024 (− 0.831; 0.783)	0.950

Analyses were performed using linear mixed-effects regression model with random intercept at visit and volunteer level. Beta represents changes in anthropometric and clinical parameters, associated with changes in gene expression levels. *p < 0.05 and ^#^p < 0.1.

## Discussion

In the present study, we assessed the utility of PBMC in humans as biological material to be used in the analysis of lipid metabolism alteration that occurs in obesity, as well as its recovery in a situation of weight loss. The possibility to have a non-invasive biological material, i.e. PBMC to deepen research on lipid metabolism is highly relevant, and it would help health professionals to determine metabolic state and the effectiveness of weight-loss interventions. We have previously demonstrated, in rodents, that obesity affects PBMC gene expression, mainly affecting lipid metabolism genes^7,9^. In the same line, here we demonstrate that OW-OB individuals present a different gene expression pattern in PBMC of key lipid metabolism genes compared to NW subjects. Higher mRNA levels of *CPT1A*, *FASN* and *SREBP-1c* genes in PBMC in the OW-OB group confirmed a clear impact of overweight and obesity on lipid metabolism, as we previously reported in rodents^7,8^. These findings also agree with those obtained by other authors in human adipose tissue and liver biopsies^29–32^. Interestingly, we found up-regulated genes from two metabolic pathways running in opposite direction. On the one hand, *CPT1A* is involved in fatty acid oxidation, while *FASN* and *SREBP-1c* play an important role in de-novo lipogenesis. It has been previously demonstrated that both pathways can operate simultaneously in order to avoid lipotoxicity and metabolic stress occurring in obesity^33^. In this sense, Lelliott and Vidal-Puig suggested that once white adipose tissue is saturated from storing triglycerides as result of a positive energy balance, other tissues, such as liver, skeletal muscle or pancreas elevate fatty oxidation pathways to compensate the increment of circulating free fatty acids^34^. However, over a long period of time, fatty acid beta-oxidation may result insufficient leading to fat deposition (by de-novo lipogenesis) in non-adipose tissues, promoting lipotoxicity and other pathologies, such as insulin resistance or fatty liver^34^. Therefore, the increased expression observed for key fatty acid beta-oxidation and lipogenic genes in PBMC of OW-OB subjects could be reflecting lipid metabolism alterations (increased fat turnover), occurring in key energy homeostasis organs to try to combat excessive fat accumulation. It should be also mentioned that several studies have reported a down-regulation of *FASN* and *SREBP-1c* in human adipose tissue of obese subjects^35–37^. Considering their increased fat mass, it may seem paradoxical to find lipogenic enzymes markedly decreased in adipose tissue. However, a different regulation pattern of de-novo lipogenesis has been reported in obesity for adipose tissue and liver: lipogenic enzymes are substantially upregulated in liver, as we observe in PBMC, but decreased in adipose tissue^38^. This behaviour could be interpreted as an adaptive mechanism of adipose tissue to prevent further fat accumulation, although the long exposure to fatty acids forces the liver to store them increasing de-novo lipogenesis. Considering these data, increased expression of lipogenesis genes observed in PBMC of the OW-OB group could be mainly reflecting the impact of increased adiposity on liver metabolism.

Interestingly, altered PBMC expression correlated directly with increased body weight, wait-hip ratio, waist circumference, % of body fat estimated using CUN-BAE and fatty liver index, as well as with circulating parameters related to obesity. For example, PBMC *SREBP-1c* expression correlated directly with fatty liver index, but also with other parameters indicative of fat accumulation in liver, such as circulating TG, CRP and GGT. In fact, subjects from the OW-OB group presented a fatty liver index score considered as suggestive of fatty liver. Moreover, in line with these observations, we observed a trend for an inverse correlation (p < 0.1) between gene expression of the lipogenic genes *FASN* and *SREBP-1c* in PBMC and lean mass. Altogether, our findings suggest that the increment in the mRNA levels of *CPT1A*, *FASN* and *SREBP-1c* displayed in OW-OB subjects is reflecting, not only accumulation or excessive fat, but also metabolic impairment related to adiposity.

Other authors have performed similar associations between altered gene expression and metabolic risk associated to obesity using tissue biopsies. For example, it has been suggested that increased expression of genes involved in both synthesis (e.g. *FASN*) and oxidation of fatty acids in subcutaneous adipose tissue of obese women is related to and contributes to insulin resistance^29^. We also obtained evidences in this sense, as we find a trend of association (p < 0.1) between *FASN* mRNA expression in PBMC and triglyceride-glucose index, considered a marker of insulin resistance.

Additional evidences of lipid metabolism impairment in overweight and obesity, which are mirrored in PBMC, are provided by a lower expression of *SCD1* and *SLC27A1* in samples from the OW-OB group observed when analysing a subset of the whole cohort. Similar results were obtained for *SCD1* by García-Serrano et al.^39^ who reported decreased mRNA levels in visceral and subcutaneous adipose tissue of subjects with morbid obesity. Regarding *SLC27A1*, while other authors have found no changes in its gene expression in adipose tissue as a consequence of obesity, a lower expression has been described for this gene in another tissue with a key role in energy metabolism, muscle, in women with obesity, as well as an inverse correlation with BMI^40^.

All in all, the use of a minimally invasive biological material, PBMC, could be helpful in the study of the pathogenesis of obesity. It is important to highlight that PBMC were able to robustly reflect an impaired lipid metabolism and increased metabolic risk in apparently healthy normoglycemic OW-OB participants. By apparently healthy we mean individuals who do not have pathological alterations in clinical outcomes (biomarkers) that normally appear when a metabolic disease is already present. This would reinforce the use of PBMC as source of early biomarkers to detect metabolic alterations before other more classical risk parameters are altered (circulating glucose, TG, etc.), helping to prevent further complications associated to overweight and obesity. It is important to remark the robustness of these observations, in spite of using young obese subjects without alteration in clinical parameters.

In addition to the previously commented genes, we also analysed PBMC expression of *PPARG*, coding for a key adipogenesis transcription factor. Overweight-obesity did not affect mRNA levels of this gene, however, sex differences were observed. NW men presented higher gene expression levels of *PPARG* than those of NW women, and the same occurred for *FASN* expression, as has been also reported in adipose tissue of rodents^41,42^.

In future studies it would of interest to perform more global approaches to obtain further insights of the usefulness of PBMC to understand the impact of increased adiposity on metabolism and health. For example, the relevance of transcriptome next generation sequencing applied to whole blood for the study of overweight/obesity and metabolic syndrome has been already established^43^. Moreover, lipidomics has been shown to detect changes in plasma lipidome with obesity and metabolic risk^44,45^. Combination of both, PBMC transcriptomics with other approaches, such as plasma lipidomics could provide a powerful tool to identify new biomarkers beyond the classic analytical parameters, as well as signatures of metabolic risk. This would be especially powerful not only for characterization of obesity, but also for diagnosis, prediction and patient personalization.

Another situation we wanted to analyse was whether PBMC were able to reflect recovery of gene expression profile as result of weight loss in the OW-OB group. In spite of obtaining a moderate decrease in body weight and fat content (4.28% and 5.12%, respectively, at the end of the intervention), metabolic recovery was evident. After 3 months of dietary intervention several parameters (circulating TG, total and LDL-cholesterol, GGT) were normalized to levels of normal-weight volunteers. This situation was maintained after 3 additional months of nutritional intervention (although no further recovery was observed). In principle, it could be expected that this metabolic improvement could be reflected into a trend to normalize the studied gene expression parameters in PBMC. However, when considering comparisons of means (repeated measures ANOVA), we only found a remarkable decrease of *FASN* mRNA levels in the OW-OB group, whose expression levels matched with those of the NW group already after 3 months of weight-loss program, but were increased again at the end of the intervention (6 months). Gene expression levels of *CPT1A* and *SREBP-1c* remained higher than in NW subjects during the whole intervention period, suggesting that they reflect deeper alterations in humans with overweight and obesity, which are not easily recovered by body fat loss. In rodents, with a more profound weight loss (approx. 20%) we have previously reported that PBMC are able to reflect nicely metabolic recovery associated to weight loss^11,13^. Therefore, our results suggest that PBMC expression of key lipid metabolism genes would be reflecting metabolic adaptations to the increased adiposity that, although decreased after the intervention period, did not reach adiposity levels of the NW group. Probably, a longer intervention period and additional weight loss would lead to a more complete metabolic normalization that could be reflected also at gene expression level when comparing group means. However, remarkably, when we used a linear mixed-effects regression model to analyse association of PBMC gene expression with the different parameters analysed, in the subjects that followed the weight-loss intervention (OW-OB, OW-OB-3M and OW-OB-6M groups), results were in concordance with those obtained at the baseline (comparing the NW and OW-OB groups). That is, considering within-volunteer’s data, increased expression of *CPT1A*, *FASN* and *SREBP-1c* correlated directly with different parameters, such as % of body fat, diastolic blood pressure, fatty liver index, total cholesterol and LDL-cholesterol, or circulating GGT levels. These observations reinforce the utility of the analysis of these genes in PBMC as indicators of fat accumulation and early metabolic alterations related to overweight/obesity, and point towards their potential interest in weight-loss studies. That is, a greater weight loss and/or metabolic improvement in the subjects participating in the intervention is associated with a lower expression of *CPT1A*, *FASN* and *SREBP-1c*. In the light of these data, further studies aimed to obtain a more consistent body weight decrease should be performed to confirm the utility of PBMC gene expression for weight loss and metabolic recovery studies. Global transcriptomic analysis in PBMC would also help to find out which metabolic pathways are involved in the health status recovery, because this is a multifactorial and multi stage process.

The main limitation of our study is the lack of tissue biopsies to check in the same volunteers that mRNA expression of the analysed genes in PBMC correlates with that in adipose tissue or liver. Tissue biopsy collection was not contemplated as our cohort was composed by young healthy adults. To solve this, we compared our gene expression results to those obtained by other researchers in adipose tissue or liver of people with overweight/obesity. Additionally, we have extensive expertise in mice showing that PBMC gene expression profile mirrors that of key energy homeostatic tissues (reviewed in^1^). Another limitation of this proof-of-concept study could be the apparent low number of subjects. However, this could be also considered as a strength, because the results obtained with a controlled number of volunteers are clear and plausible. In spite of this, it would be of interest to perform the analysis in a larger cohort to confirm and validate the utility of PBMC as a surrogate tissue for non-invasive lipid metabolism studies and maybe to find out additional correlations of interest.

In conclusion, human PBMC gene expression is affected by overweight-obesity in the same way as described in key energy homoestatic tissues. Not only this, altered gene expression in PBMC from overweight-obese individuals correlates with early metabolic alterations related to obesity. Thus, transcriptomic analysis of human PBMC constitutes a promising tool to study lipid metabolism as well as the pathogenesis of obesity without the need of performing invasive biopsies of liver or adipose tissue. Moreover, PBMC provides an interesting source of early biomarkers of impaired metabolism related to increased adiposity, e.g. (risk of insulin resistance or fatty liver), which is highly relevant to establish preventive strategies. Finally, further studies are needed to understand the usefulness of PBMC as a source of biomarkers of metabolic recovery in weight management programs.

## Supplementary Information

Supplementary Information 1.

## Data Availability

The datasets generated during and/or analysed during the current study are available from the corresponding author on reasonable request.
