# Supplementary material for: Use of human PBMC to analyse the impact of obesity on lipid metabolism and metabolic status: a proof-of-concept pilot study
Source: Sci Rep. 2021 Sep 15;11:18329. doi: 10.1038/s41598-021-96981-6 (PMC8443582; doi:10.1038/s41598-021-96981-6)
Supplement: Supplementary file 1 — Supplementary Information 1. [file 41598_2021_96981_MOESM1_ESM.pdf]

Supplementary Figure 1. Flow diagram of the progress of the trial.

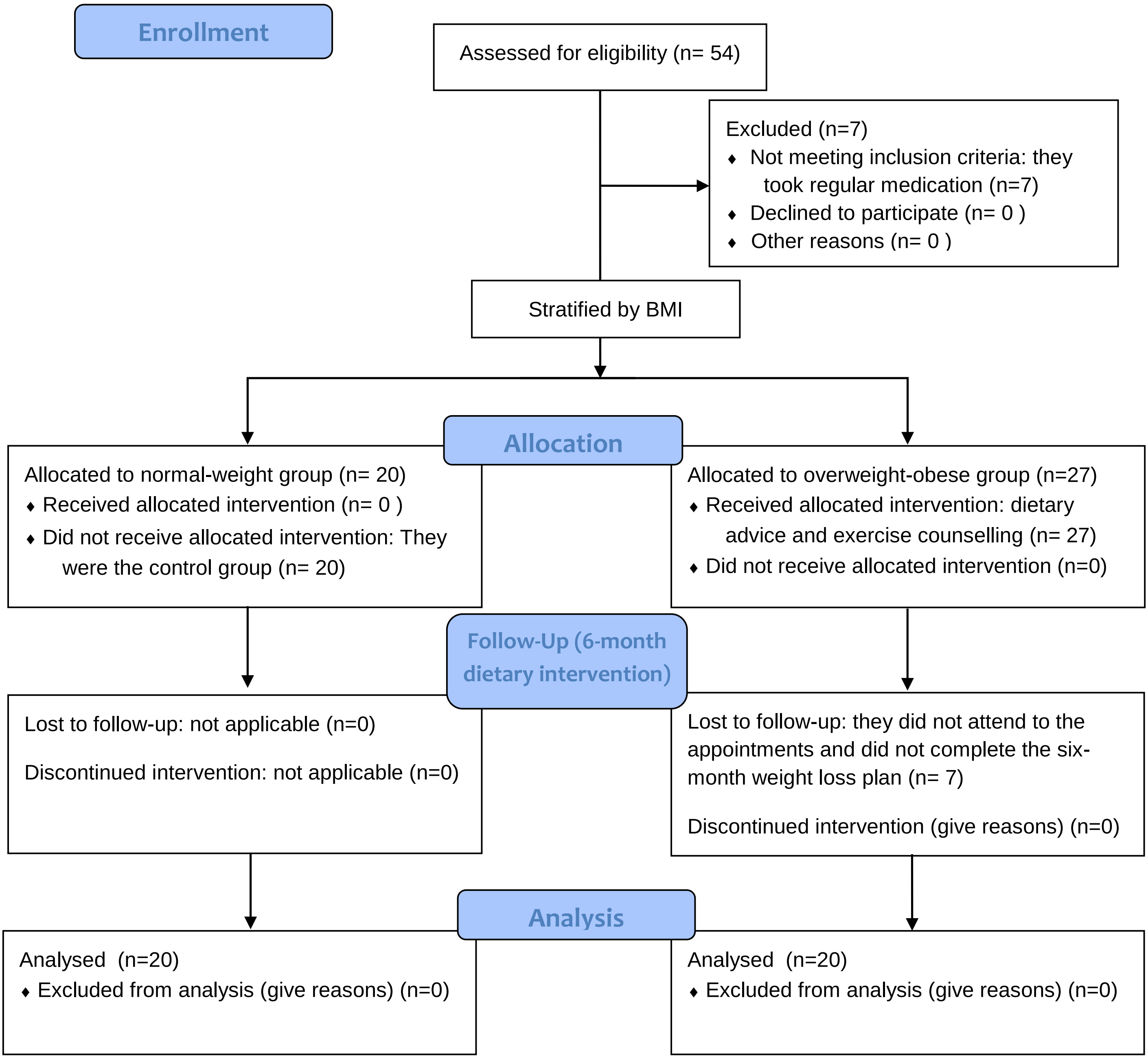

Supplementary Figure 2. Principal components analysis considering all the anthropometric and biochemical circulating parameters, as well as PBMC gene expression analysis (a total of 29 variables) for the different studied groups.

PCA considering NW and OW-OB groups

A) Score plot of PC1 versus PC2

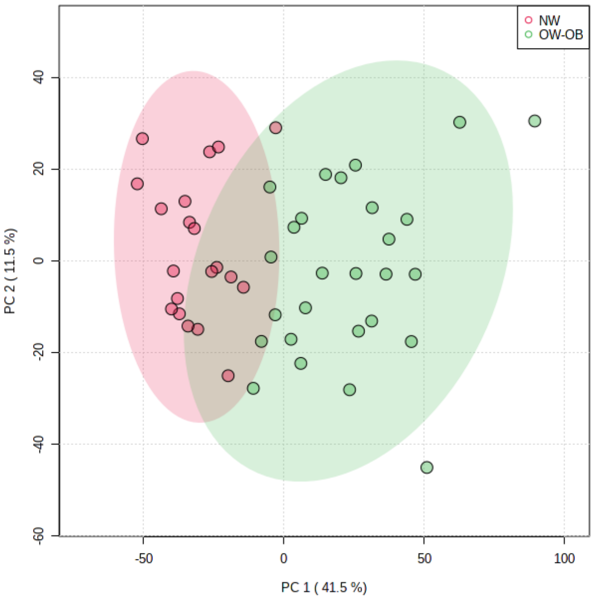

B) Contributions to the principal components (%)

**Parameter**	**PCA1**	**PCA2**
DXA (kg android fat)	0.285*	-0.100
Fatty liver index	0.271*	0.039
BMI (kg/m^2)	0.266*	-0.145
Weight (kg)	0.266*	0.045
DXA (% visceral fat)	0.260*	0.100
Insulin (µU/mL)	0.254	0.015
Android-to-gynoid ratio	0.248	0.206*
HOMA IR	0.237	0.018
Waist-hip ratio	0.224	0.401*
DXA (% body fat)	0.209	-0.443*
DXA (kg gynoid fat)	0.207	-0.415*
CUN-BAE (% body fat)	0.206	-0.300*
Lean mass (%)	-0.202	-0.056
Waist circumference (cm)	0.202	-0.056
Triglyceride-glucose index	0.187	0.120
Triglycerides (mg/dL)	0.179	0.178
LDL-C (mg/dL)	0.166	0.118
QUICKI	-0.162	0.080
Total cholesterol (mg/dL)	0.141	0.078
C-reactive protein (mg/dL)	0.109	-0.032
FASN mRNA expression	0.109	0.164
Gamma-glutamyl transferase (U/L)	0.095	0.136
SREBP-1c mRNA expression	0.089	0.112
CPT1A mRNA expression	0.073	0.131
Systolic blood pressure (mm Hg)	0.045	0.258
Diastolic blood pressure (mm Hg)	0.037	-0.003
HDL-C (mg/dL)	-0.031	-0.198
Glucose (mg/dL)	0.005	0.089
PPARG mRNA expression	0.004	0.147

PCA considering NW, OW-OB, OW-OB-3M and OW-OB-6M groups

C) Score plot of PC1 versus PC2

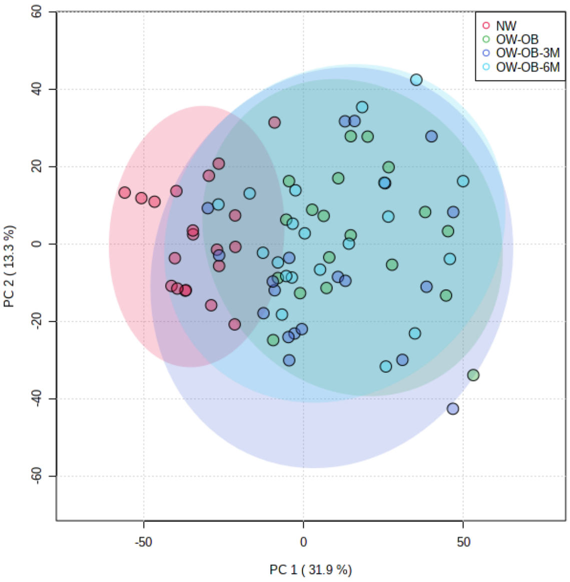

A) Principal component loadings for the first two principal components (PC); the top 5 most significant loadings are indicated with an asterisk. B) and C) Score plot representation.

Supplementary Table 1. Nucleotide sequences of primers and amplicon size used for RT-qPCR amplification in human PBMC.

**Gene**	**Forward primer (5'-3')**	**Reverse primer (3'-5')**	**Amplicon size (bp)**
ACDVL	TGCCAAGACACCAGTTACAGA	TTGAAGCCACTCCCAACCTC	207
ACSL1	TCCTTGCCCAGATGATACTTTGA	GTGGTGTTTGCTTGTCCGAA	225
CPT1A	GATTTTGCTGTCGGTCTTGG	CTCTTGCTGCCTGAATGTGA	192
DGAT1	TCCAGGGCAACTATGGCAAC	GTGTGAGGTGGCAGTGAGAA	165
FASN	GAGGAAGGAGGGTGTGTTTG	CGGGGATAGAGGTGCTGA	160
IL6	ATGTGTGAAAGCAGCAAAGAG	CACCAGGCAAGTCTCCTCAT	111
PPARG	GCCTTTTGGTGACTTTATGGA	GGCTTGTAGCAGGTTGTCTTG	175
SCD1	CCTGGTATTTCTGGGGTGAA	GGAGTGGTGGTAGTTGTGGAA	209
SLC27A1	CGCCACCAGCAAGAAGAT	GGAACAGCCACCCCATAGA	225
SREBP-1c	TGAGGACAGCAAGGCAAAG	CAGGACAGGCAGAGGAAGAC	108
TNF-alpha	TGGGCAGGTCTACTTTGGGAT	AGAGGTTGAGGGTGTCTGAA	117
Reference gene			
RPLP0	ACAACCCAGCTCTGGAGAAA	TGCCCCTGGAGATTTTAGTG	240
